# Prognostic value of tumour‐related factors associated with canine retroperitoneal hemangiosarcoma in comparison with other anatomic presentations: A retrospective observational study

**DOI:** 10.1002/vms3.1495

**Published:** 2024-06-18

**Authors:** Takayuki Furukawa, Akiko Shiotsuki, Yusami Okada, Kazumi Nibe, Meina Tei, Tetsuya Anazawa, Masakatsu Yoshikawa, Kenichiro Ono, Hidehiro Hirao

**Affiliations:** ^1^ Japan Animal Referral Medical Center (Nagoya) Nagoya‐shi Aichi Japan; ^2^ Japan Animal Referral Medical Center Kawasaki‐shi Kanagawa Japan

**Keywords:** dog, median survival time, multivariable Cox proportional‐hazard analysis, prognostic factor, retroperitoneal hemangiosarcoma

## Abstract

**Background:**

Dogs with retroperitoneal hemangiosarcoma (HSA) exhibit variable postoperative median survival times (MST).

**Objective:**

To retrospectively evaluate the prognostic value of selected tumour‐related factors, such as tumour size, rupture, invasion into adjacent tissue, involvement of lymph node and distant metastasis, they were analysed in dogs with retroperitoneal HSA.

**Methods:**

Ten dogs with retroperitoneal HSA managed solely with surgical excision were reviewed and compared with spleen (71) and liver (9) HSA. The Kaplan–Meier method and log‐rank analysis were used compare MSTs between factors. Multivariable Cox proportional‐hazard analysis was used to compare differences between arising sites.

**Results:**

Retroperitoneal HSA showed comparatively longer postoperative MST compared with that of spleen and liver HSA and demonstrated significantly longer MST (*p *= 0.003) for tumours ≥5 cm (195 days) than <5 cm (70 days). Spleen HSA revealed significantly shorter MSTs in involvement of distant lymph nodes (23 days) and distant metastasis (39 days) than those in negative (83 days, *p *= 0.002 and 110 days, *p *< 0.001, respectively). Liver HSA also revealed significantly shorter MST (16.5 days compared with 98 days, *p *= 0.003) for distant metastasis. Additionally, hazard ratios (HRs) and their forest plot for overall HSA revealed as poor prognostic factors, arising sites (spleen; HR 2.78, *p *= 0.016 and liver; HR 3.62, *p *= 0.019), involvement of distant lymph nodes (HR 2.43, *p *= 0.014), and distant metastasis (HR 2.86, *p *< 0.001), and as better prognostic factor of tumour size ≥5 cm (HR 0.53, *p *= 0.037).

**Conclusion:**

In combination with overall HSA, retroperitoneal HSA shows comparatively longer postoperative MST compared to spleen and liver HSA, associated with tumour size ≥5 cm suggesting better prognostic factor.

## Graphical abstract

To evaluate prognostic value of tumour‐related factors, including tumour size, rupture, invasion into adjacent tissue, involvement of lymph node and distant metastasis, they were analysed in retroperitoneal HSA. In combination with the results of multivariable analysis in overall HSA, retroperitoneal HSA shows comparatively longer MST, associated with the arising site and/or tumour size ≥5 cm, suggesting prognostic factors.

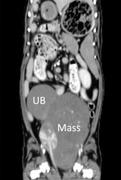

## 1 INTRODUCTION

Canine hemangiosarcoma (HSA) is the most common sarcoma, originating from vascular endothelial cells or their precursor, with typical morphological features of blood vessels (Lamerato‐Kozicki et al., 2006; Mullin & Clifford, 2019). It is often observed in aged and large‐breed dogs with no clear sex predisposition (Hirsch et al., 1981; Kim et al., 2015; Mullin & Clifford, 2020). Although it can originate in any tissue or anatomical site in the body (Griffin et al., 2021; Kim et al., 2015; Mullin & Clifford, 2019; Mullin & Clifford, 2020), it develops in the spleen in approximately half of the cases, followed by the right atrium of the heart and liver (Brown et al., 1985; Mullin & Clifford, 2020; Goritz et al., 2013). The kidney, tongue and retroperitoneal cavity are comparatively rare sites (Burton et al., 2012; Jones et al., 2007; Locke & Barber, 2006).

HSA accounts for 1%–2% of all tumours in dogs. It shows rapid growth, highly aggressive and invasive malignancy, and frequent regional and/or distal metastasis at the time of diagnosis (Mullin & Clifford, 2019; Ogilvie et al., 1996; Schultheiss, 2004). The frequent sites of HSA, particularly the spleen, reveal high malignancy and a poor prognosis (Clendaniel et al., 2014; Clifford et al., 2000; Mullin & Clifford, 2020; Griffin et al., 2021), with a high metastatic rates ranging between 16% and 89% (Batschinski et al., 2018; Moore et al., 2017; Story et al., 2020; Wendelburg et al., 2015). In contrast, the rare sites, such as the kidney, reveal a low metastatic rate (7%) (Locke & Barber, 2006). The biological behaviour of the tumour, such as the pathological future, clinical stage, tumour size, malignancy, and rupture, is considered to be closely related to the anatomical sites of the tumour (Carloni et al., 2019; Dahl et al., 2008; Griffin et al., 2021).

HSA is affected by various prognostic factors, mainly subdivided into tumour‐related, host‐related and environment‐related prognostic factors. Among them, tumour‐related prognostic factors, especially the primary tumour location, surgical resection margin and biological behaviour of the tumour (Burton et al., 2012; Carloni et al., 2019; Cospodarowicz & O'sullivan, 2003; Mullin & Clifford, 2019), are closely associated with the median survival time (MST). Postsurgical MST was reported 48–86 days for spleen HSA (Brown et al., 1985; Batschinski et al., 2018; Goritz et al., 2013; Wendelburg et al., 2015; Wood et al., 1998) and 12–17 days for right atrium HSA (Mullin et al., 2014; Yamamoto et al., 2013), 278 days for kidney HSA (Locke & Barber, 2006) and 37.5–408 days for retroperitoneal HSA (Hillers et al., 2007; Liptak et al., 2004). Retroperitoneal HSA shows variable MSTs across reports; however, information of prognostic value of tumour‐related factors is inconsistent.

The aim of the present study was to evaluate prognostic value of tumour‐related factors of retroperitoneal HSA, focusing on the biological behaviour of the tumour, especially tumour size, rupture, invasion into adjacent tissue, involvement of lymph node and distant metastasis, and to compare those with spleen and liver HSA (Burton et al., 2012; Hillers et al., 2007; Mullin & Clifford, 2019).

## 2 MATERIALS AND METHODS

### 2.1 Case selection

First, dogs with a confirmed diagnosis of HSA based on their histopathological findings were selected by two veterinary pathologists with the Japanese College of Veterinary Pathologists Board Certification from the electronic medical record database from December 2012 to July 2019. Most of the 195 dogs underwent surgical resection and were initially selected; however, 105 were excluded due to lack of surgical treatment (6 dogs), incomplete medical records (3 dogs), additional chemotherapy (70 dogs), no post‐surgical follow‐up information (17 dogs) and 9 dogs of a rare origin HSA, such as kidney (3), gastrointestinal tract (3), lymph node (1), urinary bladder (1) and vagina (1). The arising sites of HSA in the selected dogs were the retroperitoneal (*n* = 10 dogs), spleen (*n* = 71 dogs) and liver (*n* = 9 dogs). Ten retroperitoneal HSA, selected for experimental subject of this study, were clearly distinct from the kidneys, adrenal glands and ureters and urinary bladder, all of which were located in the retroperitoneal cavity. Table 1 shows the clinical information of the age (months), sex, body weight and breed (large and small) of the 90 selected dogs with HSA.

**TABLE 1:** Median survival time and *p*‐value for significance of parameters in three arising sites of hemangiosarcoma (HSA).

Arising site									
	Retroperitoneal			Spleen			Liver		
Factor	*n* = 10	MST	*p* Value^†^	*n* = 71	MST	*p* Value^†^	*n* = 9	MST	*p* Value^†^
**Age (month)**									
<120 m**onths**	4 (40%)	145		27 (38%)	57		5 (56%)	26	0.767
≥120 m**onths**	6 (60%)	213.5	0.404	44 (62%)	66	0.733	4 (44%)	88.5	
**Sex**									
Female	3 (30%)	234		26 (37%)	56		4 (44%)	97	0.501
Male	7 (70%)	155	0.744	45 (63%)	66	0.245	5 (56%)	26	
**Body Weight**									
<15 kg	7 (70%)	195		41 (58%)	76		4 (44%)	75	0.710
≥15 kg	3 (30%)	155	0.375	30 (42%)	49	0.127	5 (56%)	81	
**Breed size**									
Small	8 (80%)	165		45 (63%)	67		6 (67%)	67.5	0.305
Large	2 (20%)	193.5	0.820	26 (37%)	49	0.335	3 (33%)	98	

^†^ Log‐rank test.

### 2.2 Tumour‐related factors of HSA and follow‐up data

**FIGURE 1:**
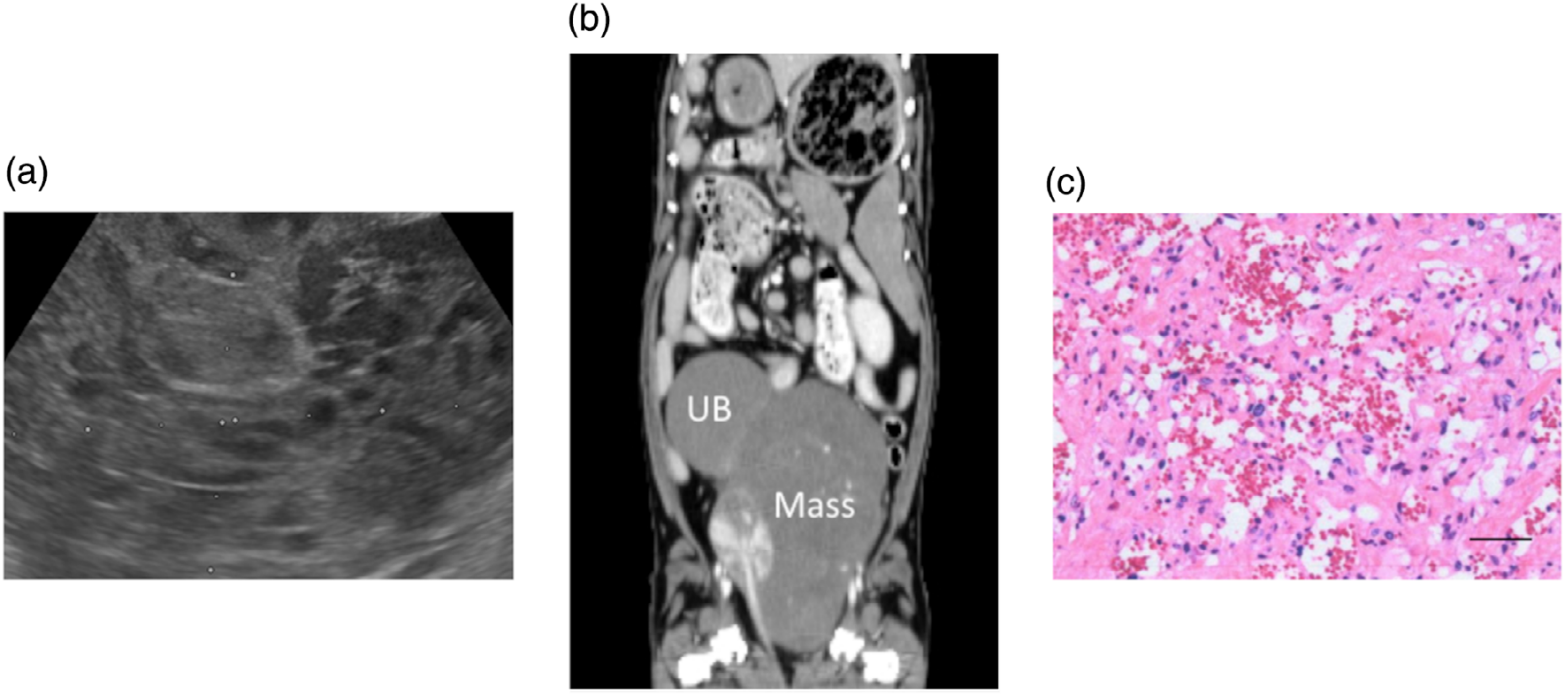
Typical ultrasonography, computed tomography and histopathological micrograph of retroperitoneal hemangiosarcoma: (a) Transverse ultrasonography reveals heterogenous hypoechoic lesions, some of which have hyperechoic rims; (b) the contrast‐enhanced dorsal computed tomography scan shows as large low‐attenuation mass in the caudal aspect of the urinary bladder; and (c) histopathological features predominantly reveal capillary growth (hematoxylin and eosin staining, bar = 100 µm).

Tumour‐related factors in the cases with HSA were as follows: tumour size, rupture, invasion into the adjacent tissues, involvement of the regional and distant lymph nodes and distant metastasis, all of which correlated closely with the prognosis of solid tumours (Burton et al., 2012; Carloni et al., 2019; Cospodarowicz & O'sullivan, 2003; Mullin & Clifford, 2019). Tumour size was calculated as the maximum length on ultrasonography (US) or computed tomography (CT). Tumour rupture was assessed on histopathology (Histo), CT, US or intraoperative findings (Ope). Invasion into the adjacent tissues was assessed on Histo, and involvements of the regional and distant lymph nodes were assessed on Histo, CT, US or X‐ray. Distant metastasis was evaluated using Histo, CT, US, X‐ray or Ope (Table 2). Typical images of US and CT and histopathological finding are shown in Figure 1.

**TABLE 2:** Distribution of predicted factors and collecting methods in three arising sites of hemangiosarcoma (HSA) in selected dogs.

		Arising site			
Factor	Collecting method^†^	Retroperitoneal (*n* = 10)	Spleen (*n* = 71)	Liver (*n* = 9)	*p* Value^‡^
**Size**					0.242
≤5 cm	CT	1	1	1	
	US	0	21	3	
		**1 (10%)**	**22 (31%)**	**4 (44%)**	
<5 cm	CT	9	8	5	
	US	0	41	0	
		9 (90%)	49 (69%)	5 (56%)	
**Rupture**					0.008^*^
(−)	Either way	7	16	2	
		**7 (70%)**	**16 (23%)**	**2 (22%)**	
(+)	Histo	2	37	4	
	CT	0	3	3	
	US	1	14	0	
	Ope	0	1^#^	0	
		**3 (30%)**	**55 (77%)**	**7 (78%)**	
**Invasion into adjacent tissue**					0.173
(−)	Histo	1	28	4	
		1 (10%)	28 (39%)	4 (44%)	
(+)	Histo	9	43	5	
		9 (90%)	43 (61%)	5 (56%)	
**Involvement of lymph node**					0.284
(−)	Either way	5	51	5	
		**5 (50%)**	**51 (72%)**	**5 (56%)**	
(+) Regional	Histo	1	3	0	
	CT	2	3	1	
	US	0	3	0	
	X‐ray	0	1	0	
		**3 (30%)**	**10 (14%)**	**1 (11%)**	
Distant	Histo	0	1	0	
	CT	2	6	3	
	US	0	2	0	
	X‐ray	0	1	0	
		**2 (20%)**	**10 (14%)**	**3 (33%)**	
**Distant metastasis**					0.863
(−)	Either way	4	31	5	
		**4 (40%)**	**31 (44%)**	**5 (56%)**	
(+)	Histo	3	19	1	
	CT	3	7	3	
	US	0	8	0	
	X‐ray	0	3	0	
	Ope	0	3^##^	0	
		**6 (60%)**	**40 (56%)**	**4 (44%)**	

Abbreviation: MST, median survival time.

^†^ CT (computed tomography), US (ultrasonography), Histo (histopathology), X‐ray and Ope (intraoperative).

^‡^ Fischer's exact test.

* Significant difference.

^#^ Bloody ascites.

^##^ Metastatic lesions in the liver.

### 2.3 Statistical analyses

Survival time was defined as the number of days from the date of surgical resection to the date of the animal's death based on medical records and available follow‐up data. MST was calculated using the Kaplan–Meier product‐limit method. Significant differences in MST among three arising sites, that is, the retroperitoneal, spleen and liver, were assessed with the log‐rank test. Using log‐rank test, significant differences in tumour‐related prognostic factors for MST were also evaluated at each arising site. As the sample size of retroperitoneal HSA was very small, Cox regression analysis was performed to obtain another supportive data. Using the multivariable Cox proportional‐hazards model and forced entry method, the covariable tumour‐related factors added to the arising site were analysed to evaluate the adjusted hazard ratio (HR) correlated with the prognosis in the overall HSA. The property of the proportional‐hazard assumption was evaluated using the Schoenfeld residual and *p*‐value. The survival package was used for the Kaplan–Meier analysis and Cox proportional‐hazards regression analysis, using R language version 3.6.3. Statistical significance was set at *p *< 0.05.

## 3 RESULTS

### 3.1 Signalment

Demographic characteristics, including age, sex, body weight and breed, were evaluated with MST and *p*‐values in three arising sites of HSA. No significant differences of parameters in each arising sites of HSA were found by log‐rank test. However, retroperitoneal HSA showed predilection of male, slightly lower body weight and high percentage of small breed (Table 1).

### 3.2 MSTs in three arising sites of HSA

**FIGURE 2:**
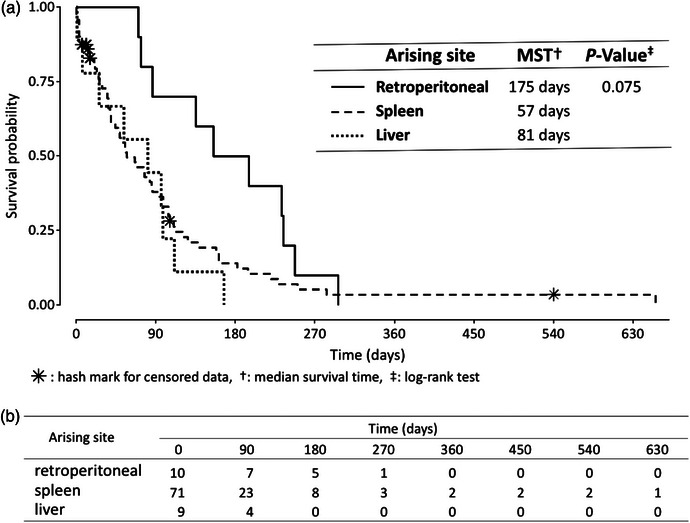
Kaplan–Meier survival curves and number at risks in dogs with retroperitoneal, spleen and liver hemangiosarcoma (HSA): (a) The Kaplan–Meier survival curves shows a median survival time of 175 days for retroperitoneal HSA, 57 days for spleen HSA and 81 days for liver HSA; (b) the number of risks in develop in the three HSA arising sites during the observation period (day 0–630).

The MSTs calculated using the Kaplan–Meier product‐limit method were 175 days (range: 70–296 days) for retroperitoneal HSA, 57 days (range: 0–655 days) for spleen HSA and 81 days (range: 1–167 days) for liver HSA. The *p*‐value (*p *= 0.075, *χ* ^2^ = 5.2, df = 2) did not differ significantly among three arising sites by the log‐rank test (Figure 2a). The numbers of events develop in the three HSA arising sites during the observation period (day 0–630) (Figure 2b).

### 3.3 Prognostic value for tumour‐related factors evaluated with MST and *p*‐value in three arising sites of HSA

Retroperitoneal HSA revealed a significantly longer MST (195 days) in tumours ≥5 cm than <5 cm (*p *= 0.003, *χ* ^2^ = 9.0, df = 1) among all potential tumour‐related prognostic factors. The remaining four factors, including rupture, invasion into the adjacent tissues, involvement of the regional and distant lymph nodes and distant metastasis, showed no significant differences of MST. In contrast, spleen HSA revealed significantly shorter MSTs if there was involvement of distant lymph nodes (23 days, *p* = 0.001, *χ* ^2^ = 10.5, df = 1 vs. negative 83 days) and distant metastasis (39 days, *p *< 0.001, *χ* ^2^ = 13.6, df = 1 vs. negative 110 days). The remaining three factors, including tumour size, rapture and invasion into the adjacent tissues, showed no significant differences of MST. In liver HSA, distant metastasis was the only tumour‐related factor associated with a significantly shorter MST (16.5 days, *p *= 0.003, *χ* ^2^ = 9.0, df = 1) than that in negative (98 days). The remaining four factors showed no significant differences of MST (Table 3).

**TABLE 3:** Median survival time and *p*‐value for significance of factors in three arising sites of hemangiosarcoma (HSA).

	Arising site					
	Retroperitoneal (*n* = 10)		Spleen (*n* = 71)		Liver (*n* = 9)	
Factor	MST	*p* Value^†^	MST	*p* Value^†^	MST	*p* Value^†^
**Size**						
<5 cm	70		57		88.5	
≥5 cm	195	0.003^*^	66	0.895	54	0.819
**Rupture**						
(−)	135		98		96	
(+)	195	0.770	56	0.266	54	0.658
**Invasion into adjacent tissue**						
(−)	135		83		75	
(+)	195	0.422	55	0.093	81	0.433
**Involvement of lymph node**						
(−)	135		83		96	
Regional	234	1.000	35	0.143	7	0.493
Distant	135	1.000	23	0.001^*^	54	0.638
**Distant metastasis**						
(−)	184.5		110		98	
(+)	175	0.965	39	<0.001^*^	16.5	0.003^*^

Abbreviation: MST, median survival time.

^†^ Log‐rank test.

* Significant difference.

### 3.4 Adjusted hazard ratios and those forest plot of prognostic factors for overall HSA

**FIGURE 3:**
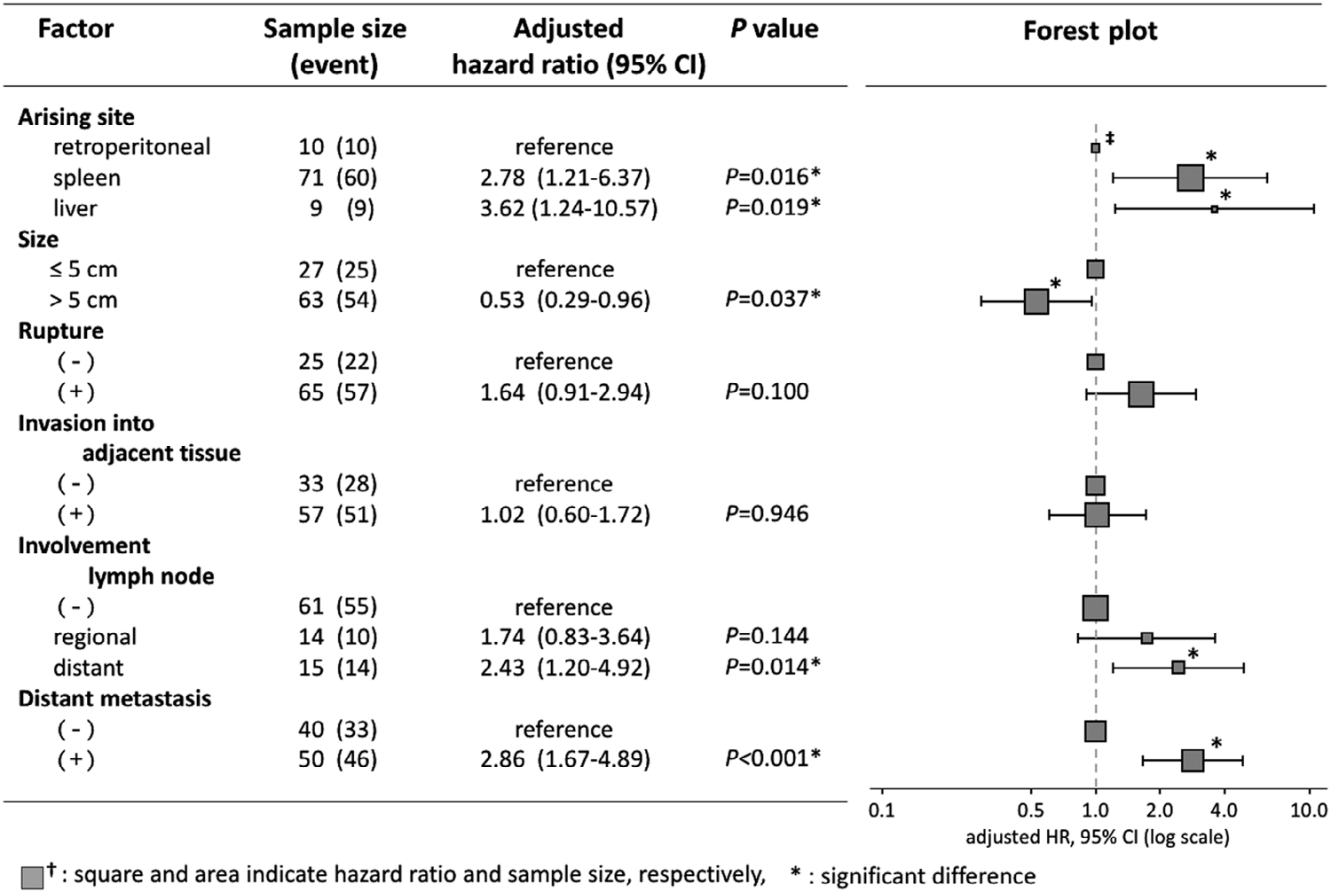
Adjusted hazard ratios (HR), 95% confidence interval (CI), *p*‐value in multivariable Cox proportional‐hazard model and those forest plots for overall hemangiosarcoma (HSA). Significantly high (1<) HRs indicating poor prognostic factors are observed for the arising sites (spleen; adjusted HR 2.78, *p* = 0.016 and liver; adjusted HR 3.62, *p* = 0.019, compared to retroperitoneal HSA), for involvement of the distant lymph node (adjusted HR=2.43, *p* = 0.014, compared to no involvement of the lymph nodes), and for distant metastasis (adjusted HR=2.86, *p* < 0.001, compared to no distant metastasis), and as better prognostic factor of tumour size >5 cm (adjusted HR = 0.53, *p* = 0.037: compared to tumour size <5 cm).

Figure 3 represents the multivariable analysis studying for difference of three arising sites together with the tumour‐related prognostic factors in overall HSA. The proportional assumptions showed no evidence of violation. Based on the adjusted HRs compared to the retroperitoneal cavity as the arising site of HSA, poor prognostic factors included spleen (adjusted HR = 2.78, 95% confidence interval [CI] = 1.21–6.37, *p* = 0.016) and liver (adjusted HR = 3.62, 95% CI = 1.24–10.57, *p* = 0.019) HSA. Other poor prognostic factors were observed, such as involvement of the distant lymph node compared to no involvement of the lymph nodes (adjusted HR = 2.43, 95% CI = 1.20–4.92, *p* = 0.014) and distal metastasis compared to no distant metastasis (adjusted HR = 2.86, 95% CI = 1.67–4.89, *p* < 0.001). However, better prognostic factor was observed in tumour size ≥5 cm compared to tumour size <5 cm (adjusted HR = 0.53, 95% CI = 0.29–0.96, *p* = 0.037).

## 4 DISCUSSION

It is well known that prognostic factors are included tumour‐related, host‐related and environment‐related factors. Although host‐related prognostic factors, such as the age of onset (≥10‐years), sex predilection (unclear) and breed‐associated predilection (large‐breed), were also affected (Hirsch et al., 1981; Kim et al., 2015; Mullin & Clifford, 2020), these distributions and differences were not so variable among the three arising sites in this study. The tumour‐related prognostic factors, such as tumour size, rupture, invasion into the adjacent tissues, involvement of the regional and distant lymph nodes and distant metastasis, were commonly used for evaluating the postoperative MST of HSAs.

In the Kaplan–Meier survival curve shown in Figure 2, retroperitoneal HSA revealed a slightly longer MST of 175 days than that of spleen (57 days) and liver (81 days) HSA. Based on the survival probability curve, spleen HSA only showed a continuous development of risk events from the middle to late follow‐up periods, similar to reported previously (Cospodarowicz & O'sullivan, 2003; Story et al., 2020). As these risk events showed non‐sporadic patterns, heterogenous and/or multiple prognostic factors might be associate with spleen HSA. The behaviour of HSA, such as the expression of cell markers, pathological features and molecular and functional subtypes, varies with the arising site (primary location) (Gorden et al., 2014; Kim et al., 2015; Lamerato‐Kozicki et al., 2006; Tomasetti & Vogelstein, 2015), and various tumour‐related factors probably affected biological behaviour.

Comparative aspect for prognostic value of tumour‐related factors was evaluated using log‐rank test in the retroperitoneal HSA and refers to those in the spleen and liver HSA. Prognostic factors were tumour size (≥5 cm) for retroperitoneal HSA with significantly longer MST, involvement of the lymph nodes and distant metastasis for spleen HSA with significantly shorter MST and distant metastasis for liver HSA with significantly shorter MST. The significant prognostic factors of retroperitoneal HSA quite differed from those of spleen and liver HSA. MST was mainly influenced by histopathological differentiation and malignancy of the original tumour for HSA (Alvarez et al., 2013; Brown et al., 1985; Batschinski et al., 2018; Dahl et al., 2008; Story et al., 2020). In 1980, the World Health Organization classified into three clinical stages: stage I, tumour size <5 cm; stage II, rupture and involvement of the regional lymph nodes; stage III, distant metastasis (Mullin & Clifford, 2019; Singer et al., 1995; Yamamoto et al., 2013). Although no clinical stage completely affected MST or prognosis of HSA (Wendelburg et al., 2015), the advanced stage/stage III was a stronger poor prognostic factor compared to stage I or II (Brown et al., 1985; Batschinski et al., 2018; Cospodarowicz & O'sullivan, 2003; Story et al., 2020; Tomasetti & Vogelstein, 2015; Treggiari et al., 2020). In the previous study on retroperitoneal HSA (Yamamoto et al., 2013), MST was significantly shorter (37.5 days) compared to this study (175 days). The prevalence rate of metastasis (89% for an MST of 37.5 days) correlates inversely with MST in comparison with 60% for an MST of 175 days. The metastasis rate was also poor prognostic factor of retroperitoneal HSA.

Furthermore, adjusted HR was calculated for the overall HSA using the multivariable Cox proportional‐hazards model. Adjusted HR suggested that one of the better prognostic factors was retroperitoneal cavity as the arising site compared to spleen (adjusted HR = 2.78, 95% CI = 1.21–6.73, *p* = 0.016) and liver (adjusted HR = 3.62, 95% CI = 1.24–10.57, *p* = 0.019). Additionally, retroperitoneal HSA showed another better prognostic factor of the tumour size ≥5 cm (adjusted HR = 0.53, 95% CI = 0.29–0.96, *p* = 0.037 compared to <5 cm). The former result is supported by the aforementioned MSTs and probably associated with a free space for growing tumour size. In contrast, tumour sizes of more or equal to 5 cm are usually associated with a poor prognosis for most tumours; however, it is not strong correlative factor affecting malignancy and/or metastasis in HSAs, particularly retroperitoneal HSA (Griffin et al., 2021; Mallinckrodt & Gottfried, 2011; Treggiari et al., 2020). In addition, both spleen and liver HSA showed significant related to the shorter MST. In this study, retroperitoneal cavity and tumour size are better prognostic factors, whereas involvements of the lymph nodes and distant metastasis are poor prognostic factors in HSAs.

## 5 CONCLUSION

In combination with the results of multivariable analysis in overall HSA, retroperitoneal HSA shows a comparatively longer postoperative MST in comparison to spleen and liver HSA, associated with tumour size ≥5 cm suggesting prognostic factor. As sample size is very small and prognostic factors are limited, further studies are necessary for confirming exact prognostic factors in retroperitoneal HSA.

## AUTHOR CONTRIBUTIONS

Kenichiro Ono and Hidehiro Hirao designed, wrote, reviewed and edited this manuscript. Takayuki Furukawa and Tetsuya Anazawa wrote original draft and analysed. Akiko Shiotsuki and Yusami Okada collected data and validated. Kazumi Nibe and Meina Tei histopathological investigated and validated. Masakatsu Yoshikawa analysed statistically this manuscript. All authors participated in the discussion and approved the final manuscript.

## CONFLICT OF INTEREST STATEMENT

The authors declare no conflicts of interest with respect to the research, authorship and/or publication of this article.

## FUNDING INFORMATION

This research did not receive any specific grant from funding agencies in the public, commercial or not‐for‐profit sectors

### ETHICS STATEMENT

All procedures involving animals were performed in accordance with the ethical standards of the institution or practice in which the studies were conducted. All animal experiments were approved by the Committee of Animal Experiments at the Japan Animal Referral Medical Center (approvable number F0602‐23‐001).

### CELL LINE VALIDATION STATEMENT

None of the cell lines were used in this study.

### PEER REVIEW

The peer review history for this article is available at https://publons.com/publon/10.1002/vms3.1495.

## Data Availability

The data that support the findings of this study are available from the corresponding author upon reasonable request.
